# New Horizons in Venous Thromboembolism Management: A Narrative Review

**DOI:** 10.3390/jcm14217668

**Published:** 2025-10-29

**Authors:** Wassim Bedrouni, Mahdi Bedrouni, James Douketis

**Affiliations:** 1 Department of Medicine, McMaster University, Hamilton, ON L8S 4L8, Canada; wassim.bedrouni@mail.mcgill.ca; 2 Department of Medicine, McGill University, Montreal, PQ H3A 3H3, Canada; mahdi.bedrouni@mail.mcgill.ca; 3 St. Joseph’s Healthcare Hamilton, Room F-544, 50 Charlton Ave East, Hamilton, ON L8N 4A6, Canada

**Keywords:** venous thromboembolism, deep vein thrombosis, pulmonary embolism, direct oral anticoagulants, factor XI(a) inhibitors, catheter-directed therapy, mechanical thrombectomy

## Abstract

Venous thromboembolism (VTE) remains a major cause of cardiovascular morbidity and mortality worldwide, and is a staple of daily clinical practice. While we have seen significant advancements in therapeutics over the last 20 years, several questions and controversies remain in the selection and duration of available therapies, as well as balancing the consequences of VTE and the bleeding risk imposed by treatment modalities. In recent years, new evidence based on randomized trials and registries have reshaped the therapeutic landscape. This narrative review synthesizes the latest advancements and future directions in VTE care, including recent guideline updates, new evidence pertaining to established pharmacologic therapy, risk stratification, interventional and procedural options, and special populations including the management of cancer-associated thrombosis, and the emerging promise of factor XI inhibition. In diagnostics, the field is moving beyond traditional methods with the investigation of novel biomarkers from proteomic and metabolomic studies and the clinical implementation of advanced imaging modalities like photon-counting CT, which offers superior resolution at lower radiation doses. Artificial intelligence is emerging as a transformative tool, potentially enhancing diagnostic accuracy in imaging. Ultimately, this review will assist clinicians in integrating evolving evidence with patient-centered decision-making to maximize benefit while minimizing harm and treating the diverse and common clinical problems of VTE.

## 1. Introduction

Venous thromboembolism (VTE), which encompasses deep vein thrombosis (DVT) and pulmonary embolism (PE), remains a source of major public health concern [1]. Incidence rates are estimated at 1–2 per 1000 in high-income countries, and age-standardized mortality rates have increased since the COVID-19 pandemic owing to vascular inflammation associated with the infection and reduced access to care during the pandemic [2,3]. Furthermore, with improved patient survival in patients with cancer, increased use of central catheters and more pro-thrombotic treatments, VTE is now the second leading cause of death after progression of malignancy [4]. Beyond the risks of the acute phase, up to 50% of patients with proximal DVT develop post-thrombotic syndrome, and 3% of patients diagnosed with acute PE progress to chronic thromboembolic pulmonary hypertension, driving a substantial quality-of-life decrement and long-term healthcare utilization [5,6].

Direct medical costs of managing VTE in the U.S. are estimated at $7–10 billion per year, driven by recurrent hospitalizations, long-term anticoagulation, and management of chronic sequelae [7]. Indirect costs of lost productivity and caregiver burden are harder to quantify but likely considerable. The economic imperative therefore aligns with clinical goals: prevent first and recurrent events while minimizing treatment-related harm.

In the past decade, treatment of VTE has shifted from vitamin K antagonists (VKAs) to direct oral anticoagulants (DOACs), alongside increased awareness and diagnosis, more refined risk stratification and management, and exploration of interventional therapies. Nevertheless, key questions remain include mitigating bleeding risk with anticoagulant therapy, identifying optimal treatment regimens for special populations including patients with cancer-associated thrombosis (CAT), and the use of catheter-based and other interventional therapies.

Against this background, this narrative review aims (i) to summarize recent evidence relating to pharmacologic advances, refinements in anticoagulant therapy, and interventional innovations for VTE, and (ii) to contextualize key randomized trials, updated guidelines, and emerging therapies when applied to clinical practice.

## 2. Evolving Landscape of VTE Guidelines in 2024

In the past year, clinical practice guideline updates from international societies have been published, reflecting the continued influx of high-quality evidence. We are observing a continued evolution towards increased granularization and complexity of VTE management, underscoring a growing need for up-to-date decision-making support tools to help clinicians.

*ASH/ISTH 2024 update: a new era for pediatric VTE treatment.* In the context of an estimated 10-fold increase in the number of children enrolled in VTE treatment trials over the intervening years, the 2024 joint guidelines from the American Society of Hematology (ASH) and the International Society on Thrombosis and Haemostasis (ISTH) represent the first major update for pediatric VTE since 2018 [8]. Although VTE incidence in the general pediatric population is low (0.07–0.14 per 10,000 children), it is 100- to 1000-fold higher in hospitalized children, making it a significant complication of complex pediatric illness.

A practice-changing shift in recent guidelines is the conditional recommendation for using DOACs, specifically dabigatran or rivaroxaban, over standard-of-care anticoagulants which include low-molecular-weight heparin (LMWH) and VKAs for the treatment of VTE in appropriate pediatric patients [8]. This key change leverages recent evidence to shift the utilization of DOACs from a secondary or off-label consideration to a preferred therapeutic option in children. Nevertheless, the panel also highlighted critical evidence gaps, emphasizing the need for more real-world data on DOAC use in diverse pediatric cohorts.

*NCCN 2024 guidelines: refining management of CAT*. For patients with cancer, VTE is a leading cause of death. Furthermore, managing the concurrent risks of thrombosis and bleeding represents a profound clinical challenge [9]. The National Comprehensive Cancer Network (NCCN) Guidelines for Cancer-Associated Venous Thromboembolic Disease (Version 2.2024) provide updated strategies for this high-risk population. The guidelines offer detailed recommendations for the evaluation and treatment of DVT, PE, superficial vein thrombosis, and splanchnic vein thrombosis. They codify the use of DOACs as a primary treatment option alongside LMWH [9]. For treatment duration, the guidelines recommend at least three months of anticoagulation, with continuation for as long as the cancer is active or the patient is receiving treatment, acknowledging the persistent prothrombotic state. Specific contraindications are also specified, including withholding prophylactic anticoagulation for platelet counts <50,000/µL.

*ESAIC 2024 update: peri-operative VTE prophylaxis*. The European Society of Anaesthesiology and Intensive Care (ESAIC) updated the 2018 peri-operative VTE prophylaxis guidelines, expanding its scope to now include urologic, plastics, and trauma surgery [10]. A central theme is the emphasis on individualized VTE risk assessment, continuing to recommend clinical prediction tools such as the Caprini score to stratify patients. For instance, in low-risk patients undergoing fast-track surgery, the guideline suggests general measures such as early mobilization and optimal hydration over routine pharmacological intervention. Conversely, for very high-risk procedures such as esophagectomy or pneumonectomy, the guidelines suggest extending prophylaxis for 28–35 days post-operatively. Recommendations are also provided for challenging populations, including obese patients (who require higher LMWH doses) and those undergoing neurosurgery or cardiac surgery, with guidance on the timing of initiation.

## 3. Innovations in VTE Diagnostics: Towards Speed and Precision

The diagnostic pathway for VTE is undergoing a technological transformation. While established clinical decision rules and imaging modalities remain foundational, recent advancements in biomarkers, imaging physics, and artificial intelligence are paving the way for a more rapid, precise, and personalized diagnostic process. These innovations aim to overcome the limitations of current tools, reduce unnecessary testing, and provide deeper insights into a patient’s specific disease state.

*Beyond d-dimer: the quest for novel biomarkers*. The d-dimer test has been a cornerstone of VTE diagnostic pathways due to its high negative predictive value in ruling out disease in low-risk patients. However, its widespread use is limited by poor specificity in patients with advanced age, cancer, or inflammation. These factors frequently lead to false-positive results and, consequently, unnecessary imaging testing [11]. This has fueled the search for novel biomarkers that more accurately reflect the specific pathophysiology of VTE and may thus provide superior diagnostic discrimination.

Recent research has focused on several promising candidate molecules. Of note, E-selectin and P-selectin, adhesion molecules implicated in critical phases of thrombus formation and inflammation, have shown potential and have been proposed as potentially offering diagnostic accuracy comparable to d-dimer but with higher specificity [12]. Recent studies have yielded mixed results, however, including a prospective observational study which found it was not useful as a marker of prognosis, namely in predicting short-term mortality in acute symptomatic PE [13]. Further research into the potential applicability of selectin molecules, particularly in the setting of CAT, where there is promise, is needed [14].

In the setting of CAT, the Khorana score is a widely used risk assessment models (RAMs) to determine VTE prophylaxis use for cancer patients starting chemotherapy but is limited by low sensitivity and specificity [15]. Management of these patients is further complicated because d-dimer levels, which are part of the RAMs are often elevated at baseline.

Ongoing research is incorporating biomarkers that reflect the unique prothrombotic state of malignancy. Studies have explored the implication of genotype in hypercoagulability and VTE, such as the presence or absence of EGFR mutation in non-small-cell lung cancer. High-throughput proteomic screens may hold the key to identifying entirely new molecular candidates and enhancing our understanding of the complex pathophysiology of thrombus [16]. Several promising biomarkers have been identified via these techniques, which can even discriminate between distinct protein expressions according to primary malignancy site and CAT, such as different profiles of immunoglobulin-derived proteins and tetranectin in lung cancer compared to pancreatic cancer patients with and without VTE [16]. Finally, a recent meta-analysis identified multiple biomarkers, including factor VIII, time to peak thrombin, fibrinogen, mean platelet volume, etc., routinely available in clinical practice which can be integrated in VTE risk models for this population and help VTE risk prediction at different stages of cancer diagnosis and treatment [17]. These tools, if clinically validated, provide an avenue for more refined thromboprophylaxis selection in a high-risk population. Ultimately, the frontier of this research likely lies in multi-omics approaches. A 2024 study utilized metabolomic profiling to identify a distinct metabolic signature in the red blood cells of patients with acute VTE, with notably three metabolites—adenosine 3′,5′-diphosphate, glutathione, and adenine—which demonstrated exceptionally high diagnostic performance, with areas under the curve (AUC) all exceeding 0.90, suggesting their potential as highly accurate, early diagnostic markers [18]. Many of these novel biomarkers, however, have yet to undergo rigorous testing in large, prospective clinical validation trials to confirm their accuracy and clinical utility [19].

*Advanced imaging modalities.* Owing to widespread availability, minimal invasiveness, and overall diagnostic accuracy, the gold standards for VTE imaging remains computed tomography pulmonary angiography (CTPA) for PE and compression ultrasound (CUS) for DVT [20,21]. CTPA has undergone advancements in recent years, as the transition from single to multi-detector CT technology has allowed for superior resolution, acquisition speed, and diagnostic accuracy, with reported sensitivity of 96%, and specificity of 98% [22]. Further, the use of dual-source scanners which utilize high-pitch scanning can reduce both contrast volume and radiation exposure, the latter being especially relevant in harm reduction in patients [20,21,22,23].

The latest technological advance is photon-counting CT (PCCT) [21,22,23]. This technology represents a fundamental change in how CT images are acquired. By directly converting individual X-ray photon energies into electrical signals, PCCT can outperform conventional energy-integrating detectors for certain tasks [22,23]. Advantages of PCCT include providing improved spatial resolution and reduces beam artifact, thereby enabling the visualization of finer anatomical details, such as the architecture of the lung parenchyma, with greater clarity. PCCT also produces superior iodine signal in vessels to conventional CT, allowing for better discrimination of small pulmonary vessel opacification, and reduces motion artifact. Perhaps most importantly, PCCT has demonstrated the ability to achieve significant radiation dose reduction of 50%, while improving image quality, as well as a reduction in contrast media, which may benefit patients with reduced renal function [22,23].

*Artificial intelligence in VTE diagnosis*. Artificial intelligence (AI), particularly machine learning (ML) and deep learning algorithms, are of particular interest for improving VTE prevention, diagnosis, and management. Currently, AI is being explored as a tool to identify at-risk patients for VTE and guiding prophylactic strategies [24]. A promising application lies in image analysis for diagnosis as CUS and CTPA have limitations related to operator dependency, false positives/negatives, and procedural risks with CTPA. AI-assisted CUS and CTA analysis, including the FDA-cleared CINA-iPE (an AI algorithm for detecting incidental PE on CTPA), have demonstrated high specificity and sensitivity [24,25]. Thus, AI could serve as a second reader for radiologists by automatically detecting PE and incidental PE on imaging studies, helping reduce missed or delayed diagnoses and improve diagnostic accuracy.

Beyond image analysis, in the face of increased imaging volumes, AI is being leveraged to optimize clinical workflows and care coordination. AI-tools which can flag suspected PE and incidental PE, triggering an alert to a multidisciplinary response team, and prioritize urgent cases on the worklist, could lead to more timely management. AI-assisted reprioritization can reduce report turnaround times by up to 12 min and cuts median detection times for incidental PE from days to just over 2 h [26]. Such AI-driven systems can ensure that high-risk patients can be prioritized to receive rapid, standardized, and coordinated care.

Despite the broad potential of AI, there are significant hurdles to its integration into clinical practice, including the need for large, diverse, and well-annotated datasets to ensure consistent performance across varied patient populations and imaging protocols. Additional challenges include inter-reader variability, data privacy concerns, lack of reimbursement models, and ethical considerations, while false positives can lead to radiologist alert fatigue, underscoring the need for careful optimization and robust IT infrastructure [26].

For all of these novel developments in VTE diagnosis, there is a need for clinical management studies that incorporate these technologies as compared to usual care before adoption into clinical practice.

## 4. Novel Paradigms in Pharmacological Therapy

Anticoagulants remain the cornerstone of VTE treatment and prevention [27]. Current limitations of available pharmacotherapies include incomplete resolution of thrombus, VTE recurrence, and bleeding. Recent research has mostly focused on DOACs in complex, high-risk and special patient populations where prior evidence from randomized trials is sparse. Concurrently, the clinical development of agents that target the intrinsic coagulation pathway is potentially opening a groundbreaking frontier, holding the tentative possibility of finally decoupling antithrombotic efficacy from bleeding risk. Recent findings from the COBRRA trial (NCT03266783), the first heard-to-head DOAC trial comparing apixaban to rivaroxaban for the treatment of acute VTE, reported a significantly lower rate of bleeding (major, clinically relevant non-major) in patients treated with apixaban [28]. These findings are consistent with findings of less bleeding with apixaban than rivaroxaban in large, linked administrative database studies involving patients with atrial fibrillation.

*Refining the role of DOACs in special populations*. DOACs are considered first-line treatment for most patients with VTE due to their convenience, effectiveness, and favorable safety profile compared to VKAs [29]. However, their optimal use in several special populations remains an area of active investigation and evolving guidance.

*Cancer-associated thrombosis*. In patients with CAT, the choice of anticoagulant is particularly complex, and is clinically important given CAT represents 30% of VTE cases [30]. Practice guidelines have increasingly supported the use of DOACs as recent trials have shown they are as effective in reducing CAT recurrence as LMWHs. The 2022 European Society of Cardiology guidelines for cardio-oncology recommend the use apixaban, edoxaban, rivaroxaban and parenteral anticoagulation for CAT (class 1, level of evidence A), while the 2021 ASH guideline suggests apixaban or rivaroxaban or LMWH for CAT as part of a conditional recommendation (very low certainty of evidence) [31,32].

A 2024 meta-analysis aimed to clarify the relative performance of each DOAC in this patient population [30]. After combining 17 RCTs with 6623 patients, the study showed no significant difference between the DOACs for the outcome of VTE recurrence, though apixaban alone demonstrated a reduced risk of recurrence as compared to parenteral anticoagulants. Additionally, apixaban was associated with a lower risk of major bleeding than edoxaban and did not increase bleeding risk compared to parenteral anticoagulants. Edoxaban was associated with a decreased risk of clinically relevant non-major bleeding as compared to rivaroxaban. This study provided the first meta-analysis to differentiate between DOACs rather than pooling them together, revealing distinct safety profiles, and only included patients with active cancer, reducing the heterogeneity of the results derived from previous meta-analyses on the topic.

Current guidelines favor LMWH in patients at risk of oral drug malabsorption, at high risk of bleeding, for example with thrombocytopenia, liver insufficiency, gastrointestinal or genitourinary tract cancers, or at risk of significant drug–drug interactions [33]. Overall, the emerging evidence further supports individualized patient management for CAT, taking into account cancer type, metastatic sites, and the safety profile of DOACs and LMWHs.

Finally, the lingering question of how long anticoagulation therapy should be pursued, and at what dose remained. In this context, the recent API-CAT trial randomized 1766 patients with CAT who had completed at least 6 months of anticoagulation to either apixaban 5 mg twice daily or 2.5 mg twice daily for 12 months [34]. It showed that the reduced-dose regimen was non-inferior in preventing recurrent VTE and was associated with a lower rate of major bleeding, which provided clinicians with key information with which to advise patients in the selection of an appropriate long-term secondary prophylaxis regimen.

*Severe renal impairment and the elderly*. Anticoagulant therapy is challenging in patients with severe chronic kidney disease (CKD), defined as a creatinine clearance (CrCl) <30 mL/min, and those with end-stage renal disease (ESRD) on dialysis. Management in this population is challenging because renal impairment is a risk factor for both thrombosis and bleeding and can affect anticoagulants that are renally cleared. Consequently, these patients were largely excluded from DOAC trials, creating a significant evidence gap [35]. Furthermore, there is a paucity of RCTs directly comparing DOACs in these patients. A meta-analysis from 2024, including data from 23 cohort studies and randomized trials and 465,673 patients with CKD, found that DOACs were associated with a significantly reduced risk of major bleeding and mortality compared to VKAs, which have been typically used in patients with atrial fibrillation or VTE and severe renal insufficiency or ESRD [36]. This provides reassurance for the use of DOACs, especially apixaban, in this high-risk group, though additional evidence is needed [37].

In elderly patients, there is hesitancy in employing advanced therapies and DOACs, borne out of concern as to the risk-benefit profile of newer treatments in the elderly [38]. Real-world data from the GARFIELD-VTE registry found that clinicians frequently opt for reduced-dose DOACs (e.g., apixaban 2.5 mg twice daily or rivaroxaban 10 mg daily) for extended secondary prophylaxis [39,40]. While the rates of VTE recurrence on these lower doses appear to be similar to full-dose therapy, the registry data reveal higher bleeding rates, likely due to the frailty and comorbidities of the patients on reduced-dose regimens which confer a higher baseline bleeding risk. There is also less use of interventional modalities in the elderly, including inferior vena cava filters, thrombolytic agents, and thrombectomy [38]. Results from a recent meta-analysis supported the use of apixaban due to a preferential bleeding risk profile [41]. Following cessation of anticoagulation therapy, these patients are at a relatively heightened risk of thrombosis recurrence and bleeding, which highlights the need for a patient-centered approach in anticoagulation selection and duration.

*Pregnancy and postpartum*. VTE occurs in approximately 1–2 out of every 1000 women during pregnancy or the postpartum period therefore detection and management is essential to reduce maternal and fetal morbidity and mortality [42,43]. The standard of care for VTE treatment and prevention during pregnancy has remained unchanged in the last year. LMWH is the anticoagulant of choice (except in patients with heparin-induced thrombocytopenia or severe renal dysfunction) due to its established safety profile and inability to cross the placental barrier. Patients with severe renal dysfunction require unfractionated heparin (UFH). VKAs and DOACs are avoided during pregnancy because they cross the placenta and have potential adverse effects on fetal development [42]. During the postpartum, LMWHs and VKAs are considered safe for use during breastfeeding. DOACs are excreted into breast milk and data on infant safety are insufficient and are thus generally not recommended. They may be considered an option only if the patient chooses not to breastfeed [43].

## 5. The Next Therapeutic Frontier: Factor XI(a) Inhibitors

The ultimate goal of anticoagulant research is to identify agents that can effectively prevent thrombosis without impairing physiologic hemostasis, thereby preventing stroke, systemic embolism and VTE while minimizing or eliminating the risk of bleeding. In this context, the factor XI (FXI) has emerged as a prime target to achieve this goal, in contrast to current anticoagulation drugs which target factor Xa or factor II (thrombin) that are further downstream in the coagulation cascade [44]. As part of the intrinsic coagulation pathway, FXI participates in amplification and stabilization of a thrombus, but has a modest role in hemostasis given most FXI-deficient patients do not experience spontaneous bleeding [45,46].

Abelacimab, a long-acting, fully human monoclonal antibody that binds to and inhibits FXI almost immediately upon infusion, has so far produced promising results [47,48]. In a phase 2 trial involving patients who had total knee arthroplasty, a single post-operative intravenous dose of abelacimab, 75 mg or 150 mg, was statistically superior to enoxaparin 40 mg, by reducing the rate of VTE by approximately 80% with no observed bleeding [47]. In the recent AZALEA-TIMI 71 phase 2 trial, which compared once-monthly subcutaneous abelacimab to rivaroxaban or stroke prevention in atrial fibrillation, was terminated early due to a greater-than-expected reduction in clinical bleeding [49]. The 150 mg dose of abelacimab reduced the rate of major or clinically relevant non-major bleeding by 67% and the rate of major bleeding alone by 74% compared to rivaroxaban.

These preliminary findings are promising and suggest that FXI(a) inhibitors, with a seemingly significantly superior bleeding risk profile, could represent the most significant paradigm shift in anticoagulation management since the advent of DOACs. Presently, Phase 3 trials, including ASTER and MAGNOLIA, are ongoing to evaluate abelacimab for the treatment of CAT. Patients with CAT may particularly benefit from an anticoagulant with a more favorable bleeding profile, though they are also at high recurrent VTE risk and these trials will be a major test of the antithrombotic capacity of abelacimab [27].

Other novel pharmacotherapies in development are antagonists of fibrinolysis inhibitors, including α2-antiplasmin and thrombin-activatable fibrinolysis inhibitors, which may dissolve venous thrombus without significantly increasing bleeding risk, by enhancing natural clot-dissolving mechanisms [27,50,51,52]. Phase 2 trials of these agents are summarized in Table 1.

## 6. Advancements in Interventional Management of Acute VTE

The management of intermediate-risk PE occupies a clinical gray zone, with physicians balancing the potential for hemodynamic deterioration on anticoagulation alone, against potentially exposing the patient to the significant bleeding risks of full-dose systemic thrombolysis. Practice guidelines generally recommend anticoagulation for patients with intermediate-risk PE, based on high rates of intracranial hemorrhage (3–4%) associated with systemic thrombolysis. Mortality rates remain elevated in these patients, emphasizing the need for alternative therapeutic options to improve outcomes [53]. Catheter-based therapies emerged as a potential middle-ground solution, but data was derived from observational studies, and direct comparisons were lacking until recently. Advancements in endovascular device technology and the recent data from randomized trials, summarized in Table 2, have the potential to affect management of high-risk acute VTE.

*PEERLESS trial: interventions for intermediate-risk PE*. This trial, which enrolled 550 patients, was the first large-scale randomized trial to compare two leading catheter-based interventions: large-bore mechanical thrombectomy (LBMT) with the FlowTriever system and catheter-directed thrombolysis (CDT) [53]. The primary endpoint was a hierarchical win ratio composite of all-cause mortality, intracranial bleeding, major bleeding, clinical deterioration, and post-procedural ICU admission and length of stay. The results appeared to favor LBMT, suggesting that patients are five times more likely to avoid one of the primary endpoints with LBMT [54]. This difference between the treatment arms was not driven by differences in mortality or major bleeding, which were low and similar between groups, but by significant reductions in downstream resource use and clinical events. Patients receiving LBMT had less post-procedural ICU utilization (41.6% vs. 98.6% required ICU admission) and significantly fewer episodes of clinical deterioration or need for rescue therapy (1.8% vs. 5.4%). LBMT also was associated with shorter total hospital stays and fewer 30-day readmissions. It should be noted that the inclusion of ICU utilization as part of the primary outcome was criticized, as centers almost universally treat patients who undergo CDT in the ICU, and its exclusion would prevent the primary outcome from achieving statistical significance. Nevertheless, the PEERLESS results support LBMT as the preferred interventional strategy over CDT, though in centers with requisite expertise and in appropriately selected patients with intermediate-risk PE, demonstrating that faster, more complete thrombus removal leads to more rapid clinical improvement and lower resource utilization.

*HI-PEITHO trial: addressing a key question in PE management*. While the PEERLESS trials sought to answer the question of which catheter-based intervention is superior, it did not address the question of whether to intervene at all in this patient population, in the absence of an anticoagulation-only control arm. The ongoing HI-PEITHO trial is addressing this issue by comparing ultrasound-assisted, catheter-directed thrombolysis (USCDT) plus standard anticoagulation versus anticoagulation alone in patients with intermediate-high-risk PE [55]. The primary outcome is a composite of PE-related death, cardiorespiratory decompensation, or recurrent PE within 7 days of randomization. A positive result for the USCDT would provide high-quality evidence to support upfront interventional therapy over a conservative approach in this population. Conversely, a neutral result would reinforce the current strategy of anticoagulation first, with intervention reserved for patients who deteriorate on anticoagulation.

*Interventional Strategies for DVT*. In contrast to PE, where intervention is often aimed at preventing immediate cardiorespiratory collapse, one of the primary goals of intervention in DVT is to reduce the long-term burden of post-thrombotic syndrome (PTS), a potentially disabling consequence and condition characterized by pain, swelling, skin changes and potentially skin ulcers [56]. Prior enthusiasm for intervention was tempered by the ATTRACT trial, which concluded that for most patients with proximal DVT, adding CDT to anticoagulation did not reduce the overall incidence of PTS [57]. The follow-up CAVA trial failed to demonstrate a reduction in PTS in the treatment arm with CDT over anticoagulation only for patients with iliofemoral DVT [57].

On the other hand, subgroup analysis of the ATTRACT trial demonstrated that patients with iliofemoral DVT may benefit from a greater reduction in moderate-severe PTS incidence compared to anticoagulation alone [58]. As a result, guidelines recommend CDT for patients with extensive iliofemoral DVT, particularly when associated with phlegmasia cerulea dolens, while select guidelines such ASH recommend it for younger patients with a low bleeding risk, in whom reducing the severity of PTS is a key therapeutic goal [58].

Finally, interventional modalities for VTE treatment continues to advance, with a range of mechanical thrombectomy devices now available, including large-volume aspiration systems (Penumbra Indigo, AngioVac), rotational systems (Cleaner), and mechanical coring and extraction systems (ClotTriever), each with unique design features optimized for removing organized thrombus from the venous system [58,59,60].

## 7. Advances in VTE Prevention and Risk Stratification

Recent advancements in this are transitioning from the one-size-fits-all model of risk stratification toward more dynamic and personalized prediction methods. This shift aims to improve the identification of high-risk patients who need prophylaxis and to recognize low-risk individuals who can safely avoid unnecessary treatment.

*Limitations of traditional risk assessment models (RAMs)*. Clinical guidelines have supported the use of validated VTE risk assessment models (RAMs) such as the IMPROVE and Padua scores for hospitalized medical patients and the Khorana score for ambulatory cancer patients [61,62]. However, a 2025 study compared six RAMs (Khorana, PROTECHT, CONKO, COMPASS-CAT, CATScore, and EHR-CAT) in cancer patients, reporting that all the models displayed poor to modest predictive performance, highlighting a limited predictive ability, in part due to an inadequate capture of VTE risk associated with cancer treatments [63].

Another challenge in predicting risk of VTE to guide thromboprophylaxis is the need to balance VTE risk against bleeding risk. Anticoagulant thromboprophylaxis is highly effective at reducing the incidence of VTE but is associated with an increased bleeding risk, in addition to increasing healthcare costs. Although multiple VTE RAMs exist, validated bleeding RAMs for medical inpatients have been scarce which led to the development and validation of the Cleveland Clinic Bleeding Model (CCBM), which incorporates 16 factors (e.g., active peptic ulcer disease, prior bleeding, and sepsis) and appears to be better able to discern in-hospital bleeding compared to the IMPROVE bleed risk model [64].

*Towards dynamic and personalized risk prediction*. Researchers are increasingly turning to ML models for generating more precise and targeted de-escalation strategies. However, these models face their own unique sets of challenges. Due to the low incidence rate of VTE, there are significantly fewer VTE patients compared to non-VTE patients. This gap leads to a class imbalance issue, a common issue in machine learning wherein one class in a classification problem significantly outweighs the other class which results in inferior and unstable model performance. A 2024 study sought to overcome this challenge in order to develop a more accurate ML VTE risk model [65]. The authors defined and modeled a “fuzzy population” of patients who share identical risk profiles but belong to different outcome groups (VTE vs. non-VTE). Using this method, they successfully developed a model that achieved a higher specificity and an equivalent sensitivity as compared to the traditional Padua [65]. This study underscores the unique challenges faced by ML models but also their vast potential to generate more robust, precise, and clinically meaningful risk prediction tools.

Identifying high-risk patients is not the only goal of creating more precise risk prediction models. Indeed, these VTE prevention strategies are also aimed towards sparing low-risk individuals from unnecessary treatment. In this respect, the 2024 TriP(cast) trial used a score to stratify VTE risk based on trauma type, immobilization, and patient characteristics [66]. The score was used to safely identify patients with lower limb trauma who did not need preventive anti-coagulants. The strategy serves to reduce burden, cost, and potential harm to patients who can forgo anti-coagulant treatment without increasing the risk of VTE [66]. Thus, developing these models fulfills the pressing need for personalized, risk-adapted prevention that minimizes overtreatment while preserving safety.

The goal of personalized prophylaxis is RAMs that identify high-risk patients, while safely excluding low-risk patients. The future of VTE prevention could very well involve a two-step assessment. A scoring system such as the TriP(cast) can be used to safely exclude the majority of low-risk patients from pharmacological intervention. An additional step could involve a more personalized assessment that integrates specific risk factors such as bleeding risk to quantify their net clinical benefit and guide the decision-making. Taken together, the aim would be to minimize errors of omission whereby patients did not receive thromboprophylaxis when indicated) and commission (patients did receive thromboprophylaxis when not indicated.

## 8. Future Directions and Unmet Needs

By enabling the integration of novel diagnostic technologies, the management of VTE is shifting towards more personalized patient management and away from traditional broad, one-size-fits-all approaches This transition will enable more reliable diagnoses, safer therapeutics, and evidence-based interventions. However, despite this positive inflection point, significant challenges remain including resolving the data gaps that exist for understudied patient groups and the management of chronic VTE sequelae.

Generating personalized VTE prevention and treatment is central to the future landscape of VTE management. This will require the integration of comprehensive risk models that combine genomic, proteomic, and metabolomic data with dynamic clinical variables and AI-enhanced imaging. Thus, clinicians may be able to generate a comprehensive, real-time, and accurate risk profile for both thrombosis and bleeding and allow personalized thromboprophylaxis [3]. In addition, the emergence of factor XI(a) inhibitors have the potential for improved safety profiles but requires comparable efficacy to DOACs, which remain as first-line anticoagulants for multiple preventive and therapeutic clinical indications [27].

In the interventional space, the future lies in delineating the patient populations that would benefit from endovascular treatments, as well as the appropriate selection of available modalities. With high-quality data from the PEERLESS trial and forthcoming results from HI-PEITHO, improved evidence-based pathways for managing intermediate-risk acute PE will emerge. Within a decade, this may be supplemented by further integration and elaboration of the role of multidisciplinary VTE response teams, which may leverage AI-powered triage and integrated risk assessment to rapidly select the most appropriate therapy for each patient, whether anticoagulation alone, large-bore mechanical thrombectomy, or systemic thrombolysis.

Despite this progress, several unmet needs persist, including questions as to the optimal prevention and treatment of chronic VTE sequelae, PTS, and chronic thromboembolic pulmonary hypertension. While acute therapies have improved, they have had a limited impact on these long-term complications. Furthermore, high-quality evidence is needed to guide VTE management in special populations that are often excluded from major trials, including patients with severe obesity, severe renal or hepatic failure, and during pregnancy. Finally, the challenge of clinical translation cannot be overstated. The development of new diagnostics and therapeutics is only effective if they are integrated equitably, efficiently, and accurately into clinical practice.

## 9. Conclusions

The landscape of VTE diagnostics and management is rapidly transforming. In response to the wave of AI technologies, diagnostic and risk assessment tools that leverage the power of this burgeoning field in addition to state-of-the-art imaging innovations and multi-omics are being developed to provide more comprehensive and quantitative insights into disease prevention and management. Most significantly, the development of factor XI(a) inhibitors has the potential to refine pharmacological therapies. Finally, clinicians will have the ability to select patients with intermediate-risk PE for appropriate catheter-based therapies based on high-quality randomized trial data. The field of VTE management is shifting towards more personalized prevention and treatment strategies that consider an individual’s unique risk profile based on clinical, molecular, and imaging data. Although some challenges are yet to be satisfactorily addressed, notably in preventing and treating the chronic sequelae of VTE, the future of VTE management is progressing toward one where patients receive increasingly safer and more effective treatment.

## Figures and Tables

**Table 1:** Emerging and Investigational Antithrombotic Agents for VTE.

Agent Name	Target/Class	Mechanism of Action	Key Clinical Trial (Year) & Findings
**Abelacimab**	Factor XI/XIa Inhibitor	Prevents activation of Factor XI, inhibiting the intrinsic coagulation pathway while preserving hemostasis.	**AZALEA-TIMI 71 (2025):** In AF, reduced major/CRNM bleeding by 67% vs. rivaroxaban.
**Osocimab**	Factor XIa Inhibitor	Directly inhibits activated Factor XIa.	**FOXTROT (2020):** Non-inferior to enoxaparin/apixaban for VTE prevention post-TKA.
**Milvexian**	Factor XIa Inhibitor	Directly inhibits activated Factor XIa.	Phase 3 trials ongoing; represents an oral option in this class.
**DS1040**	TAFI Inhibitor	Inhibits Thrombin-Activatable Fibrinolysis Inhibitor, promoting natural clot breakdown.	**Phase 1b PE Trial (2023):** Safe but no significant benefit in reducing thrombus burden in intermediate-risk PE.
**TS23/BAY3018250**	α2-Antiplasmin Inhibitor	Inhibits the primary inhibitor of plasmin, enhancing fibrinolysis.	**NAIL-IT/SIRIUS (2023/2024):** Phase 2 trials recruiting for PE and DVT; represents a novel pro-fibrinolytic approach.

**Table 2:** Landmark and Ongoing Interventional Trials for Acute PE.

Feature	PEERLESS Trial	HI-PEITHO Trial
**Status**	Completed (Results presented 2024)	Ongoing (Recruiting)
**Population**	Intermediate-risk PE	Intermediate-high-risk PE
**Intervention**	Large-Bore Mechanical Thrombectomy (FlowTriever)	Ultrasound-Assisted Catheter-Directed Thrombolysis (EKOS)
**Comparator**	Catheter-Directed Thrombolysis (CDT)	Anticoagulation Alone
**Primary Endpoint**	5-component hierarchical win ratio (mortality, ICH, major bleed, clinical deterioration, ICU use)	7-day composite of PE-related death, cardiorespiratory collapse, or recurrent PE
**Key Finding/Question Addressed**	**Finding:** LBMT is superior to CDT, driven by less ICU use and clinical deterioration.	**Question:** Is USCDT + Anticoagulation superior to Anticoagulation alone?
